# Post Mortem DNA Degradation of Human Tissue Experimentally Mummified in Salt

**DOI:** 10.1371/journal.pone.0110753

**Published:** 2014-10-22

**Authors:** Natallia Shved, Cordula Haas, Christina Papageorgopoulou, Guelfirde Akguel, Katja Paulsen, Abigail Bouwman, Christina Warinner, Frank Rühli

**Affiliations:** 1 Institute of Evolutionary Medicine, University of Zurich, Zurich, Switzerland; Department of Forensic Genetics, Institute of Legal Medicine, University of Zürich, Zürich, Switzerland; 3 Division of Cell- and Neurobiology, Institute of Anatomy, University of Zurich, Zurich, Switzerland; Kliniken der Stadt Köln gGmbH, Germany

## Abstract

Mummified human tissues are of great interest in forensics and biomolecular archaeology. The aim of this study was to analyse post mortem DNA alterations in soft tissues in order to improve our knowledge of the patterns of DNA degradation that occur during salt mummification. In this study, the lower limb of a female human donor was amputated within 24 h post mortem and mummified using a process designed to simulate the salt dehydration phase of natural or artificial mummification. Skin and skeletal muscle were sampled at multiple time points over a period of 322 days and subjected to genetic analysis. Patterns of genomic fragmentation, miscoding lesions, and overall DNA degradation in both nuclear and mitochondrial DNA was assessed by different methods: gel electrophoresis, multiplex comparative autosomal STR length amplification, cloning and sequence analysis, and PCR amplification of different fragment sizes using a damage sensitive recombinant polymerase. The study outcome reveals a very good level of DNA preservation in salt mummified tissues over the course of the experiment, with an overall slower rate of DNA fragmentation in skin compared to muscle.

## Introduction

Mummification is the process by which soft tissue decomposition after death is halted or significantly slowed, resulting in long-term preservation. From a medical perspective, mummies enable the origins and evolution of human disease to be studied directly, thereby making them a unique reservoir of information about the human past [1], [2]. In most forms of mummification, soft tissue is preserved when tissue dehydration slows or halts postmortem decay. This may be achieved either naturally, as a result of environmental conditions, or it may be induced by intentional human practices that result in artificial mummification.

Natural mummification can occur under a variety conditions. Examples of natural mummification by desiccation are typically found in hot and arid environments, such as Egypt [3], Nubia [4], the Canary Islands [5], and sometimes in combination with cold arid plateaus, such as in northern Chile/southern Peru [6]–[8]. Ötzi, a Neolithic Iceman found in a glacier in the Italian Alps, is an example of exceptional mummification resulting from desiccation in extreme cold [9], [10]. Mummification can also be facilitated in temperate environments with the addition of a chemical desiccant, such as salt. The burial of a body within salt deposits, as occurred during a series of cave-ins at the Chehr Abad salt mines in Iran from 500 BC to AD 500, can result in natural salt mummification [11]–[13] .

Natural mummification can also occur under wet conditions such as with the Mawangdui female corpse of Changsha, China [14] and the bog bodies of northern Europe [15]. Anoxic liquid environment, acidic water, lack of accessible nitrogen, sequestering essential metal cations and low temperature inhibit aerobic microbial growth [16]. In northern European peat bogs, the chemical sphagnan, a polysaccharide compound produced by sphagnum mosses, imparts additional antimicrobial activity to the water which further inhibits aerobic microbial growth [17], and also further promotes soft tissue preservation through spontaneous Maillard reactions with ammonia and amino acids in the bog [17], [18] .

Examples of artificial mummification are known from both ancient Egypt and the Chinchorro culture of South America. During ancient Egyptian artificial mummification water was removed from the body using natron, a mixture of salts composed of sodium carbonate, sodium bicarbonate, sodium sulphate and sodium chloride. After desiccation, the body was then embalmed with resins and oils that chemically stabilized the tissues and further inhibited decay [4], [19], [20]. The Chinchorro culture of southern Peru and northern Chile also prepared their dead using a form of artificial mummification. Though considerable variation occurred over the several thousands of years that the Chinchorro culture persisted, fundamental body treatments for a significant portion of the deceased involved defleshing, cleaning, and wrapping the bones with fiber, subsequently replacing the skin. The artificial body thus created was frequently covered with clay and painted with pigment [7], [18], [21], [22].

Although mummification can be described in broad terms, many aspects of the process remain poorly characterized and are only partially understood. Natural mummification is often a sporadic occurrence, and preservation can vary greatly both among and within individuals. During natural mummification by desiccation, for example, it is not uncommon for a head or a foot to preserve exceptionally well while the rest of the body is reduced to mere skeletal remains. By contrast among bog bodies, the skin and hair is often preserved in exquisite detail but the entire skeleton may be lacking if the pH of the bog is low. In addition to tissue-level differences, variation can also be observed in the preservation of biomolecules, such as DNA. Although ancient DNA can be extracted from Peruvian mummies or permafrost mummies like Ötzi, DNA recovery from ancient Egyptian mummies is more controversial [23]–[25], and bog bodies almost always fail to produce authentic ancient DNA [26].

Teasing apart the various factors that cause these differences is a difficult challenge, and relatively few studies have specifically investigated experimental mummification in chickens and pigeons [27]–[29], rabbits and fish[30], mice and rats [31], dogs [32], ducks [33], sheep [34] and humans [20], [35], [36]. Moreover, occasional mummification experiments on single organs, e.g., modern placenta [37] have been made. In most cases of mummification there are multiple factors operating simultaneously (temperature, humidity, pH, antimicrobial chemical agents, etc.), sometimes in conjunction and at other times in opposition.

In order to better characterize the mummification processes, we have isolated a crucial step common to several forms of artificial and natural mummification, salt desiccation, and replicated it under laboratory conditions. Natural salt mummies have been found from collapsed salt mines in Cherabad, Iran [11] and in areas of high salt deposits near Hallstatt, Austria [18].

The study of post mortem DNA damage during the mummification process is critically important to ensure the generation of accurate data from ancient or forensic sources of DNA. The aim of this research is to characterize the effect of salt desiccation on the preservation of human tissues at the biomolecular level. This is the first study to apply molecular methodologies to the investigation of post mortem alterations in a human experimental mummification setting.

## Materials and Methods

### Experimental design and tissue sampling

The following artificial mummification experiment was carried out in the Institute of Anatomy at the University of Zurich in accordance with medical ethics standards and permissions, including an *intra vitam* body donation declaration. Written informed consent from the donor was obtained by the Institute of Anatomy for use of this sample in research. Ethics committee approval of Canton Zurich was not required for this research project, since research projects on body parts of deceased donors, who donated their bodies during their lifetime to the Institute of Anatomy, University of Zurich for research projects do not require additional approval of the ethics committee (cf. §29 Abs.5 as well as §32 Patients law), and a letter of waiver was written to this effect.

A lower limb (LL) was amputated from an adult female donor 24 h post mortem (day 0) who had died of natural causes. The LL was desiccated in a container of natron salt, in simulation of the desiccation phase of an ancient Egyptian mummification. For our experiment 70 kg of artificial natron was prepared. Prior to use, the components of the artificial natron (Table 1) were purchased (Sigma-Aldrich), powdered and baked at 100°C overnight and then mixed according to Brier and Wade [36]. A layer of natron approximately 10 cm thick was placed below the LL in a pine box and the remaining natron was used to cover it. The natron-covered LL was kept in a chemical fume hood under constant ventilation. Temperature, relative humidity, and pH were continuously monitored throughout the experiment. The pH of the LL was measured at two sites: the proximal femur amputation site, and the mid-calf sampling area.

**Table 1:** Natron composition.

Salt	%
NaCl	53.81
Na_2_SO_4_	15.91
Na_2_CO_3_	18.26
NaHCO_3_	12.03

Skin and muscle tissue samples were collected from the mid-calf sampling area at days 1, 3, 5, 7, 11, 14, 19, 25, 32, 38, 45, 52, 60, 73, 94, 125, 160, 208, 322 . Each tissue sample was separated into three parts, one to assess histological and the remaining two for assessing biomolecular post mortem changes. Following each tissue collection, the LL was transported to University Hospital Balgrist, Zurich for MRI and CT imaging to further characterize tissue changes. The LL was then replaced in the natron mixture. Tissue samples for genetic analysis were shock frozen in liquid nitrogen and stored at −80°C until analysis. For standard histological analysis, tissues were fixed in 4% paraformaldehyde, dehydrated, embedded in paraffin wax and sectioned using microtome (Microm HM 325, Adamas Instrumenten,). Routine histological staining (Hematoxylin-Eosin) was performed as discussed in another publication [38].

### DNA extraction and quantification

Genomic DNA was extracted in the Institute of Anatomy, University Zurich, twice for each time point from both skin and muscle samples using a QIAamp^R^ DNA mini extraction kit (Qiagen CA) following manufacturer protocols. Between 25 and 45 mg of each tissue was used in the extraction. Quantification of extracted DNA from each sample was performed using a NanoDrop ND-1000 spectrophotometer and a Qubit fluorometer (Invitrogen).

### DNA fragmentation assessment

#### a) Gel electrophoresis

Whole genomic DNA was extracted from paired skin and muscle samples, in duplicate on separate occasions, and fragmentation was assessed using two methods. First, genomic DNA fragmentation in skin and muscle samples was visualized using gel electrophoresis by loading 5 µL DNA extract into a 0.8% agarose gel containing Gel-Red DNA stain and characterizing genomic DNA fragmentation across multiple time points.

DNA fragmentation analysis was also performed using the Agilent DNA 12000 kit (PN 5067-1508) on the Agilent 2100 Bioanalyzer System (Agilent Technologies) following the manufacturer's protocols. The 12000 kit can measure DNA fragments up to 12,000 bp long and has the highest upper size limit among the Agilent kits.

#### b) Autosomal STR amplification

To characterize DNA fragmentation at a sub-genomic level on a scale most relevant for forensic and ancient DNA applications (50–400 bp) approximately 1 ng DNA was amplified with the SGM Plus multiplex kit (Applied Biosystems/Life Technologies) in a total reaction volume of 25 µL on a GeneAmp PCR System 9700 (Life Technologies) according to the manufacturer's protocol. PCR products were detected with a Genetic Analyzer 3130 xl (Life Technologies). One microliter of the amplified sample was then added to 12.5 µL Hi-Di Formamide and 0.5 ml of GeneScan-500 ROX (Life Technologies). The following electrophoresis conditions were used: 18 s injection time, 1.2 kV injection voltage, 15 kV run voltage, 60°C, 25 min. run time, Dye Set F (5-FAM, JOE, NED, ROX). Raw data were analyzed with the Genemapper Software (Life Technologies). A peak detection threshold of 50 RFUs was used.

### Cloning and sequencing/Damage detection

To assess changes in DNA sequence quality (i.e., nucleotide lesions) during salt mummification, 1000 bp long nuDNA and mtDNA targets spanning identity informative regions (mitochondrial HVRI and autosomal STR D18S51) were PCR-amplified from skin and muscle samples from day 0 and day 322. Each 25 µL reaction contained 1× AmpliTaq Gold buffer (Life Technologies), 1.5 mM MgCl_2_ (Life Technologies), 0.5 µM forward and 0.5 µM reverse primers (Microsynth), 0.2 mM each dNTP, 0.25 µL AmpliTaq Gold, and 10 ng template DNA. The PCR cycling conditions were as follows: 95°C for 10 min, 30 cycles of 95°C for 30 s, 58°C for 30 s, and 72°C for 30 s, and a final extension at 72°C for 10 min. Table 2 and Table 3 show the primer sets used to amplify a product of 999 bp for nuDNA and of 1019 bp for mtDNA. All PCR products were cloned by inserting the amplicons into pBluescript KS vectors (Stratagene), followed by transformation into competent *Escherichia coli* cells using standard protocols. Between 20 and 25 colonies from each sample were randomly picked and PCR amplified with Go Taq Flexi DNA Polymerase (Promega) in 25 µL reaction volume. Each reaction contained 1× Go Tag Flexi buffer (Promega), 0.5 µM T3 forward (ATTAACCCTCACTAAAGG) and 0.5 µM T7 (AATACGACTCACTATAGG) reverse primers (Microsynth), 0.2 mM each dNTP, 0.2 µL Go Taq Flexi DNA Polymerase. The PCR cycling conditions were as follows: 95°C for 2 min, 25 cycles of 95°C for 20 s, 50°C for 20 s, and 72°C for 30 s with final extension at 72°C for 10 min.

**Table 2:** Nuclear DNA primers.

STR Locus	Primers (5′- 3′): F - forward, R - reverse	Amplicon size (bp)	Source
D18S51	F: CAAACCCGACTACCAGCAAC R: AAAGGGGAAGCAGCTCAAGT	999	Oldroyd, 1995 This study
D18S51	F: CAAACCCGACTACCAGCAACR: TATGATTGTCAGGGGACATGG	1951	Oldroyd, 1995 This study
D18S51	F: CAAACCCGACTACCAGCAAC R: TCTAGAAGCCAGTGTCCCTCA	3953	Oldroyd, 1995 This study
D18S51	F CAAACCCGACTACCAGCAACR: ACCACTTTTGGAAACTCTCTGG	7936	Oldroyd, 1995 This study

**Table 3:** Mitochondrial DNA primers.

MtDNA (rCRS)	Primers (5′- 3′): F - forward, R - reverse	Amplicon size (bp)	Source
mtDNA	F: GCACCCAAAGCTAAGATTCTAATTT R: GGGGTGACTGTTAAAAGTGCATAC	1019	Kemp 2007
mtDNA	F: GCACCCAAAGCTAAGATTCTAATTT R: CCTTAAGTTTCATAAGGGCTATCG	1991	Kemp 2007 This study
mtDNA	F: GCACCCAAAGCTAAGATTCTAATTT R: CGTCAGCGAAGGGTTGTAGT	4040	Kemp 2007 This study
mtDNA	F: GCACCCAAAGCTAAGATTCTAATTT R: GTTCTTCGAATGTGTGGTAGGG	8005	Kemp 2007 This study

Positive PCR reactions were sequenced using a Genetic Analyzer 3100 (Life Technologies) and analyzed using CLC Main Workbench software. The resulting sequences were aligned against the mtDNA and nuDNA sequences obtained at day 0 to analyze for postmortem damage sites. All day 0 mtDNA sequences were aligned and compared to the Cambridge reference sequence (rCRS; GenBank:NC_012920)[39], [40] and day 0 nuDNA sequences were aligned to the human reference including reference sequence for commonly used STR marker D18S51 (GenBank:AP001534). Consensus sequences were generated by aligning all replicates and clones from each day and identifying common polymorphisms. Unique polymorphisms (i.e., substitutions that were present in only one or a few clones) were then regarded as potential damage sites. Since each cloned target spanned an identity informative region, sources of non-endogenous contamination could be identified and excluded, thereby assuring confident characterization of endogenous post mortem of miscoding lesions.

### Analysis of DNA damage: Phusion amplification of multiple target lengths

Overall DNA damage (fragmentation and base modifications) in skin and muscle samples was assayed by PCR amplification using Phusion polymerase (Thermo Scientific), a highly accurate and thermostable recombinant DNA polymerase that stalls at most miscoding lesions, such as cytosine deaminations and abasic sites [41]. Four overlapping primers sets in both the nuclear and mitochondrial genomes targeting sequences of increasing length from 1000–8000 bp were PCR-amplified (Tables 2 and 3) for all sampled days post-mortem. The targets, which span the mitochondrial HVRI and the autosomal STR marker D18S51, were chosen to provide identity information that could be used to detect and exclude possible contamination. Each 20 µL reaction contained 1× Phusion HF buffer (Finnzymes), 0.5 µM forward and 0.5 µM reverse primers (Microsynth), 0.2 mM each dNTP, 0.25 µL Phusion DNA polymerase and 2 µL DNA extract (5 ng/µL). The PCR cycling conditions were as follows: 98°C for 30 s, 30 cycles of 98°C for 10 s, 66–68°C for 20 s, and 72°C for 30–240 s (product length-dependent), and a final extension at 72°C for 10 min. All amplified products were first visualized by agarose gel electrophoresis and then sequenced and compared to the LL day 0 consensus sequences in order to exclude potential non-endogenous amplifications.

### Statistical analyses

Chi-square statistics with Yates correction (χ2) was performed (GraphPad InStat3) to investigate whether the observed patterns of base transitions and transversions in mtDNA and nuDNA sequences were similarly distributed. All tests were performed at the 5% significance level.

## Results

### Macroscopic and microscopic characteristics of the mummified tissues

At day 322 of the mummification experiment the foot of the LL was substantially shrunken and dehydrated, whereas the thigh had not yet reached the complete dehydration and mummification. The weight of the LL decreased from 6.049 kg on day 0 to 3.232 kg on day 322 of the mummification experiment. During the experiment, the temperature fluctuated from 20–26°C and relative humidity ranged from 35–70%. The pH of the LL was observed to increase from pH 6 to 10 over the course of the experiment.

**Figure 1:**
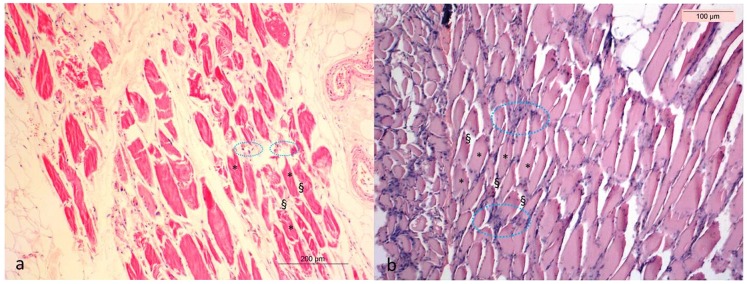
Hematoxylin-Eosin (a, b) stained section of muscle tissue from the lower limb at Day 3 (a) and Day 208 (b). The muscle fibres (+) after 208 days are shrunken but morphologically unchanged. The nuclei (circled in blue) of the muscle cells are clearly stained until the end of the experiment. The epimysium (§) shows minimal changes.

The histological examination reveals generally good soft tissue preservation. The muscle fibres were shrunken, but otherwise morphologically unchanged. The epimysium, connective tissue that surrounds the muscle fibres, showed minimal alteration. The nuclei of the muscle cells stained clearly for the entire duration of the experiment. The skin was similarly well preserved, although structural changes were more pronounced compared to muscle. By day 208, the five layers of the epidermis were no longer easily distinguishable: the dermis was shrunken, and structures such as the vascular networks of the papillary dermis and the free sensory nerve endings, were not discernible (Figure 1) [38].

### DNA fragmentation assessment

#### a) Gel electrophoresis

**Figure 2:**
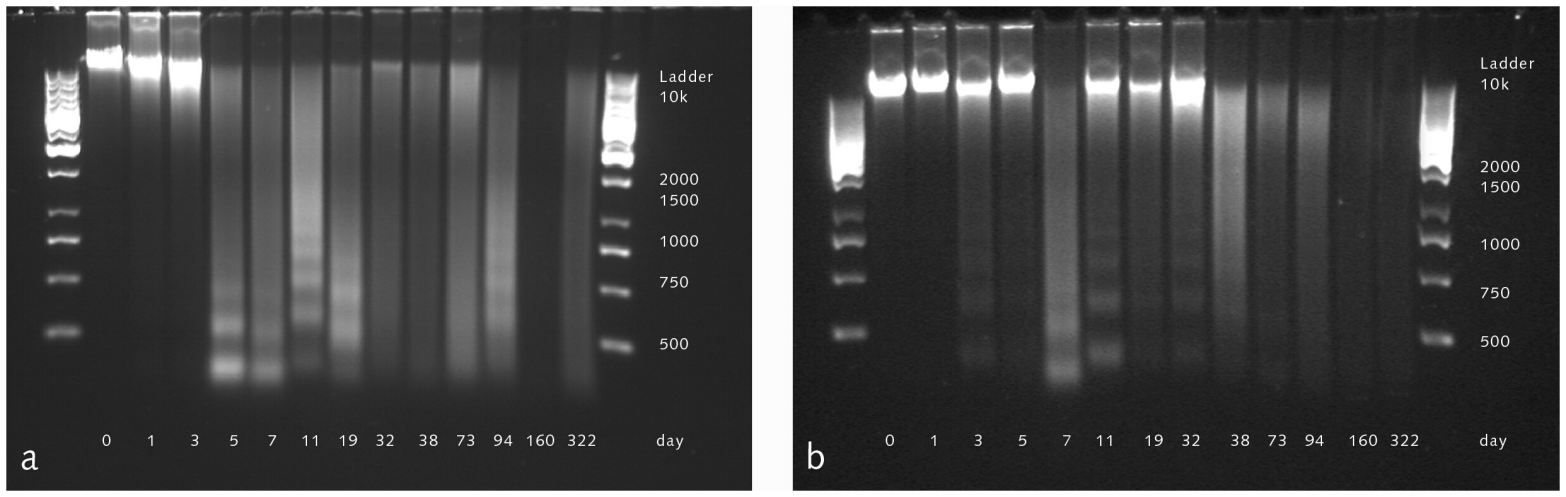
The fragmentation of genomic DNA was investigated by 0.8% agarose gel electrophoresis. High molecular weight, genomic DNA can be seen in muscle extracts up to day 3 postmortem (a) before fragmentation leads to band smearing. This process occurs much later in skin, where high molecular weight genomic DNA is not observed after 32 days postmortem (b).

Whole genomic DNA fragmentation in muscle and skin extracts from multiple time points was visualized using gel electrophoresis (Figure 2). High molecular weight genomic DNA can be seen in skin extracts up to day 32 before fragmentation leads to band smearing. Genomic DNA fragmentation was observed much earlier in muscle, where band smearing began as early as day 5. Observation of DNA fragments using the Bioanalyzer system was not possible because DNA fragment length exceeded the size limit of the assay (data not shown).

#### b) STR analysis

In order to evaluate viability of DNA amplification at a sub-genomic level on a scale most relevant for forensic and ancient DNA applications (50–400 bp), DNA was amplified using the AmpFlSTR SGM Plus PCR Amplification Kit, a multiplex STR assay commonly used in forensic analysis (Table 4). The kit simultaneously tests for variable length polymorphisms ranging in size from approximately 100–350 bp at 11 independent loci throughout the nuclear genome. In cases of poor DNA preservation, as is common in forensic and ancient DNA samples, the longer amplicons may fail to amplify (allelic dropout) as DNA becomes increasingly fragmented. The artificially mummified leg, however, exhibited no allelic dropout of STR markers up to 350 bp. Additionally, identical results were obtained for each time point, which confirms the lack of contamination and robust quality of the DNA in both tissues.

**Table 4:** Results of SGM Plus AmpFlSTR genetic typing.

Locus designation	Alleles range	Skin		Muscle	
		Day 0 Alleles	Day 322 Alleles	Day 0 Alleles	Day 322 Alleles
Amelogenin	XX, XY	XX	XX	XX	XX
D19S433	9–17.2	13, 14	13, 14	13, 14	13, 14
D3S1358	12–19	15, 18	15, 18	15, 18	15, 18
D8S1179	8–19	13	13	13	13
VWA	11–24	16	16	16	16
TH01	4–13.3	7	7	7	7
D21S11	24–38	30, 31	30, 31	30, 31	30, 31
FGA	17–51.2	24, 25	24, 25	24, 25	24, 25
D16S539	5–15	11, 12	11, 12	11, 12	11, 12
D18S51	7–27	15	15	15	15
D2S1338	15–28	17, 25	17, 25	17, 25	17, 25

**Figure 3:**
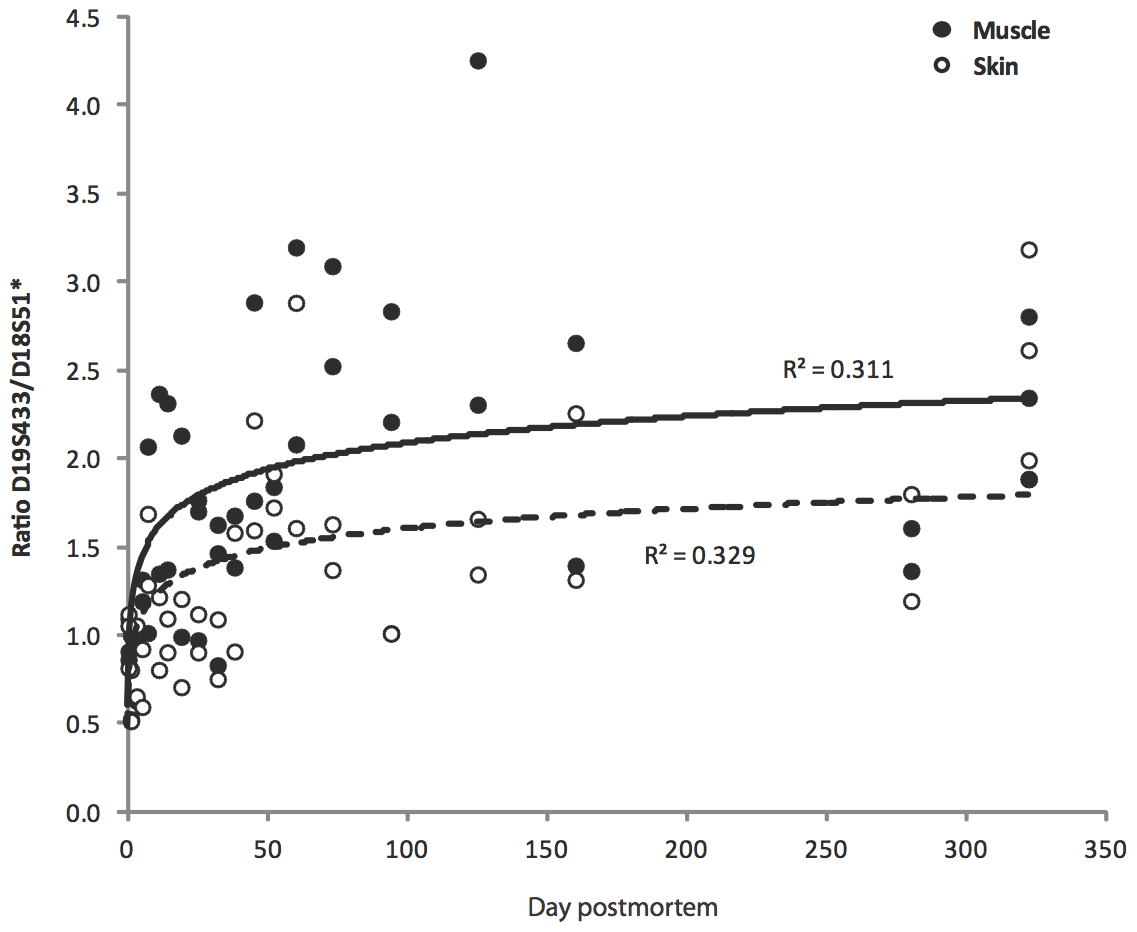
The change of the peak height ratio of the shorter D19 locus (sum of the peak heights of alleles 13 and 14) relative to the longer D18 locus (peak height of homozygous allele 15) within the DNA profiles of muscle and skin samples during the 322 days of salt desiccation. For both tissues, the line of best fit for the change in the peak height ratio through time is logarithmic, with lower ratios observed for skin.

In the multiplex STR assay, signal intensity is proportional to the quantity of DNA containing the targeted marker. In fresh tissue, the quantity of DNA should be equal for all markers, but in highly degraded tissues, DNA fragmentation results in an underrepresentation of longer markers. In order to compare the relative proportion of short to long targets, a ratio was calculated for the shorter locus D19S433 (amplicon range 102–135 bp) and the longer locus D18S51 (amplicon range 262–355 bp) (Figure 3) [42]. A ratio of 1 indicates no DNA size bias in the extract. A ratio greater than 1 indicates an excess of short targets in the DNA extract, likely caused by the loss of longer DNA targets through fragmentation. At day 0 of the artificial mummification experiment the ratio is approximately 1, but this value increases to over 2 for both skin and muscle tissue by day 322.

### Damage detection

In this study, nucleotide damage was assessed by multiple sequence alignment of 137 sequences from two time points (day 0 and day 322 post mortem) and two tissues (skin and muscle). All mtDNA and nuDNA sequences were aligned to the revised Cambridge Reference Sequence (rCRS) [39], [40] and human reference sequence D18S51 locus, (GenBank AP001534), respectively, and the diagnostic mitochondrial SNPs and autosomal STR repeats were used to authenticate the endogenous origin of the DNA and exclude contamination. Nucleotide variations compared to the donor's consensus were analyzed as a potential damage sites. Table 5 provides the total number of cloned and sequenced bases per sample, the number of damaged bases observed within each time point, and *χ^2^* values for comparison between days 0–322. No significant differences were observed in mitochondrial DNA between day 0 and 322 in any tissue. The same is true for nuclear DNA damage in skin. Slight, but non-significant, differences, however, were observed in muscle nuclear DNA (Table 5). The miscoding lesions were then categorized by DNA damage type (Table 6) according to Hansen et al. [43].

**Table 5:** Post mortem single-base damages.

Tissue	DNA	Days post mortem	Total bases^a^	Damaged bases^b^	Damage per base^c^	?^2^ *p* value
**Skin**	mitochondrial	0	12868	3	2.33E-04	
	mitochondrial	322	13225	4	3.02E-04	0.7325
	nuclear	0	17021	3	1.76E-04	
	nuclear	322	16285	4	2.46E-04	0.9534
**Muscle**	mitochondrial	0	15104	3	1.99E-04	
	mitochondrial	322	17261	5	2.90E-04	0.8686
	nuclear	0	14224	2	1.41E-04	
	nuclear	322	13223	8	6.05E-04	0.0895

a Total number of bases cloned and sequenced per sample.

b Total number of observed damage events per sample.

c Number of damage events per nucleotide position.

**Table 6:** Post mortem base substitutions by type.

Tissue	DNA type	Day	Individual base substitution												Transitions		Transversions	
			AG	TC	GA	CT	AC	TG	AT	TA	CA	GT	CG	GC	Type1	Type2	Type1	Type2
**Skin**	Mt	0	0	2	0	1	0	0	0	0	0	0	0	0	2	1	0	0
	Mt	322	0	0	2	2	0	0	0	0	0	0	0	0	0	4	0	0
	Nu	0	1	1	0	0	0	0	1	0	0	0	0	0	2	0	1	0
	Nu	322	0	3	0	1	0	0	0	0	0	0	0	0	3	1	0	0
**Muscle**	Mt	0	0	0	1	2	0	0	0	0	0	0	0	0	0	3	0	0
	Mt	322	1	1	1	0	0	0	0	0	1	0	0	1	2	1	0	2
	Nu	0	0	0	0	2	0	0	0	0	0	0	0	0	0	2	0	0
	Nu	322	2	3	0	3	0	0	0	0	0	0	0	0	5	3	0	0

### Assessment of DNA damage by PCR amplification with Phusion DNA polymerase

In order to characterize overall DNA damage, we next amplified the DNA extracts for mitochondrial and nuclear DNA targets of four lengths (1000, 2000, 4000, and 8000 bp) using Phusion, a synthetic DNA polymerase that is highly sensitive to many forms of DNA damage. Unlike Taq-based polymerases, as was used in the AmpFlSTR assay and to characterize miscoding lesions in amplicon clones, Phusion DNA Polymerases have extremely low error rates (4.4×10^−7^). The error rate, determined by a modified lacI-based method [44] , is approximately 50-fold lower than that of Taq DNA polymerase. Additionally, Phusion DNA Polymerase is tolerant of many inhibitors and other challenging reaction conditions, thereby minimizing reaction failures. However, Phusion polymerase is highly sensitive to DNA damage and stalls at a wide range of miscoding lesions [41]. As a result, it detects a wider range of DNA damage and is a good test of overall biomolecular degradation during salt mummification.

**Figure 4:**
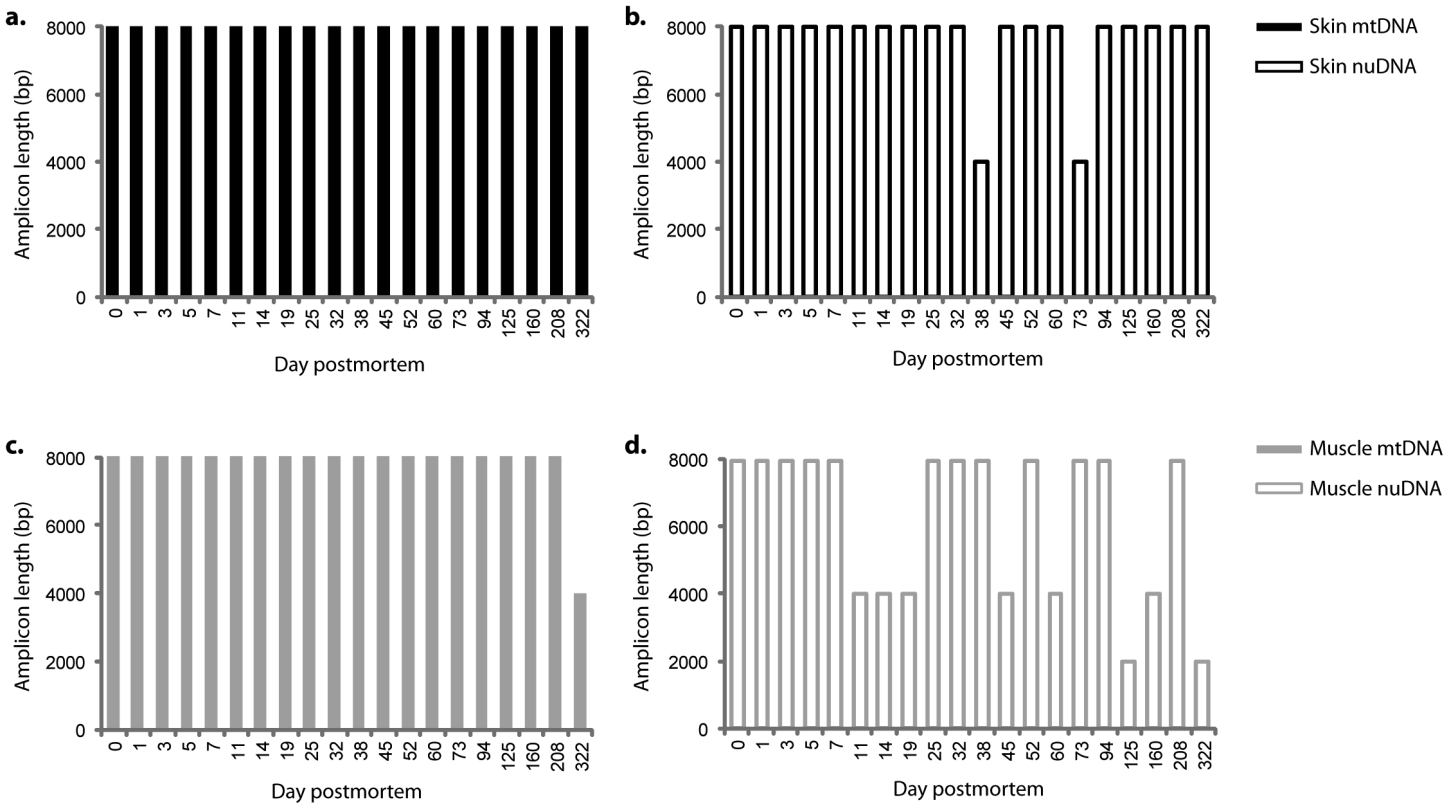
Maximum target size amplification of (a, c) mitochondrial (mt) and (b, d) nuclear (nu) DNA from both (a, b) skin and (c, d) muscle tissue extracts from day 0 to day 322. Skin (black line), Muscle (gray line), mtDNA (solid line), nuDNA (dashed line).

Long (8000 bp) mitochondrial and nuclear targets could be readily amplified from the day 0 extracts (Figure 4). Over time, the maximum amplifiable target length generally decreased, with earlier declines in muscle than in skin. Longer targets also failed to amplify earlier in nuclear DNA than in mitochondrial DNA, as is expected, since the copy number of mitochondrial genomes is on the order of thousands per cultured mammalian cell [45]. However, as can been seen in Figure 3, damage-free 4000 bp nuclear targets and 8000 bp mitochondrial targets were readily amplifiable from the mummified skin tissues, and 2000 bp nuclear targets and 4000 bp mitochondrial targets were amplifiable from the muscle samples almost one year post-mortem. To confirm that these results are not the result of contamination, the amplicons were sequenced and aligned to the DNA sequence of the leg at day 0. The specimens exhibited identical sequence polymorphisms in the mitochondrial HVRI and autosomal STR marker D18S51, confirming that the amplifications are endogenous and authentic.

### Statistical analyses

Although we observed slightly greater nucleotide damage at day 322 than at day 0, none of these increases are significant (Table 5). In addition, although we consistently observed significant differences between transversions and transitions (χ^2^ test P = 0.0265 – <0.0001) across the date points and tissues (Table 7), we found no significant difference between type 1 transitions (A→G; T→C) and type 2 (C→T; G→A) transitions: p = 0.8527 (total type 1 vs. type 2); p = 0.7518 (day 0 type 1 vs. type 2); p = 0.8185 (day 322 type 1vs. type 2).

**Table 7:** Results of χ^2^-test.

Test		?^2^ value	*p*
**Total**	TS vs. TV	19.534	<0.0001
Skin	TS vs. TV	8.644	0.0033
Muscle	TS vs. TV	9.390	0.0022
**Day 0**			
Total	TS vs. TV	5.819	0.0159
Skin	TS vs. TV	1.500	0.2206
Muscle	TS vs. TV	3.200	0.0736
**Day 322**			
Total	TS vs. TV	12.193	0.0005
Skin	TS vs. TV	6.126	0.0133
Muscle	TS vs. TV	4.924	0.0265

The number of damage events in different tissues and at different time points (skin and muscle, day 0 and day 322) is compared.TS =  transitions; TV =  transversions.

## Discussion

Forensic and ancient DNA is often highly degraded. Cell rupturing and the subsequent release of nucleases and other DNA damaging chemicals causes oxidative and hydrolytic damage to DNA, resulting in DNA fragmentation, base alteration, cross-linking, and other forms of degradation and damage. As a result, ancient DNA fragments rarely exceed 500 bp in length and are characterized by a high frequency of miscoding lesions and abasic sites [46], [47]. During the mummification process, DNA templates also can become highly fragmented or chemically modified, therefore reducing the quantity and quality of DNA templates and consequently leading to PCR failure, allelic dropout, and miscoding lesions. In order to characterize the effects of salt desiccation on the DNA degradation process, artificially mummified skin and muscle samples were evaluated using several different approaches for measuring DNA fragmentation and damage.

In our study we simulated the natron salt desiccation step of ancient Egyptian mummification described by Herodotus (5^th^ c. BC), modified to extend the desiccation period from 40 days to 322 days [18]. We found that following 322 days of natron desiccation the LL appeared mummified and lacked gross evidence of severe decay, even though dehydration was incomplete in the LL interior muscle groups. We attribute this lack of dehydration to the unstable and relatively high humidity inside the hood and the lack of dry air circulation. The amputated leg lost approximately 50% of its weight over the course of the experiment, and the skin was observed to darken considerably, likely as the result of melanin concentration during epidermal dehydration. A steady increase in pH was measured over the experimental period, which likely resulted from the release of ammonia during chemical or microbial degradation of nitrogenous matter during putrefaction [48]–[50]. Histological comparison of skin and muscle samples revealed surprisingly good overall structural preservation. Although shrunken, the muscle fibres at the end of the experiment were morphologically unchanged, and skin was similarly well preserved, although structural changes were more pronounced compared to the muscle. In both skin and muscle samples, numerous cell nuclei remained visible up to day 322 of the experiment, indicating the long-term preservation of relatively intact cells [51].

After death, DNA rapidly begins to decay, leading to forms of damage such as inter- and intramolecular crosslinking, strand fragmentation, abasic sites and modified nucleotides [52]. Analysis of genomic DNA preservation by electrophoresis indicates that high molecular weight genomic DNA is lost earlier in muscle than in skin, a finding that likely results from the fact that skin is in direct contact with natron salt, and therefore is more efficiently dehydrated. Indeed, upon visual inspection, the skin appears dry, while interior muscles are visibly damp. Thus, these results indicate that thorough desiccation is a key component to slowing DNA fragmentation.

Autosomal STR analysis supports these findings (Figure 3). By comparing the signal intensities of shorter and longer targets (D19S433/D18S51 ratio), a fragment ratio can be calculated. A fragment ratio of 1 indicates no size bias in the DNA extracts. The fragment ratio of both skin and muscle samples increased from 1 on day 0 to >2 on day 322, indicating 50% excess of short DNA fragments under 300 bp in length. As with the electrophoresis results, this effect was more prominent in muscle than in skin samples. Nevertheless, even the day 322 samples (skin and muscle) exhibited no allelic dropout of STR markers up to 350 bp, which indicates sufficient DNA preservation for most forensic DNA applications nearly one year post mortem. In a future study, later time points during mummification could be analyzed in order to determine when DNA fragmentation prevents STR profiling.

We evaluated DNA fragmentation of two tissues, muscles and skin, by targeting four different mitochondrial and nuclear fragment sizes. Post mortem age varied between 0 and 322 days. We used a Phusion High-Fidelity DNA Polymerase which sensitive to any type of DNA damage and as a result it detects not only DNA fragmentation but also a wider range of DNA lesions. Phusion DNA Polymerase is tolerant of many PCR inhibitors and provides reliability by minimizing reaction failures, and enables PCR from unpurified sample materials. In addition, Phusion DNA Polymerases have extremely low error rates (0.11%) confirmed by studies using 454 sequencing and Illumina sequencing [53] . PCR can routinely amplify DNA from extracts containing as few as 10–100 template copies [54]. In order for PCR to succeed using Phusion, there must, therefore, be at least 10–100 damage-free template copies per aliquot of extract used in the PCR reaction. The failure of a PCR reaction to amplify indicates that there were fewer than 10–100 damage-free target molecules per 10 ng aliquot of extract.

Although next generation sequencing (NGS) can be a powerful tool for evaluating DNA fragment length in highly degraded samples, NGS cannot be used to assess preservation in samples this well preserved. The observed fragment lengths in this study were so long that they would have required shearing prior to NGS analysis, a step that would have defeated the goals of this damage assessment. For this reason, NGS was not attempted.

The PCR amplifiable level of each amplicon was assessed in comparison with the level at day 0. These data in turn enabled two complementary analyses to be performed, tracking of each target through the mummification time and the differential degradation between the four targets. These two measures therefore enable the assessment of the rate of degradation of single transcript and the relative rate of degradation of multiple transcripts, respectively. As expected in the beginning of the experiment all mitochondrial and nuclear targets could be successfully amplified from both tissues. But over time, the maximum amplifiable target length decreased, with earlier decline in muscle (day 11 post mortem) than in skin (day 38 post mortem). Nuclear DNA also failed to amplify earlier than mitochondrial, which is in agreement with experience on aDNA analysis [55].

Analysis showed that post mortem DNA degradation was present in both muscle and skin tissues. The types of damage identified include DNA fragmentation, nucleotide substitution and DNA loss. This resulted in band smearing on gel electrophoresis and increased ratio of shorter to longer fragments in STR analysis. In addition, the frequency of successful PCR of longer nuclear and mitochondrial PCR products declined earlier than for shorter products over time. These changes were first observed in muscles and then in skin, which can be due to the direct proximity of skin to the salt and thus the higher efficiency of dehydration of skin than muscles.

We used a non-proofreading Taq (AmpliTaq Gold) to identify nucleotide miscoding lesions. The degree of nucleotide damage we observed was consistently low across both targets and both tissues. Although we observed more degradation in day 322 samples than the day 0, none of these increases are significant (Figure 4). This indicates that under salt dessication conditions, at least in the first year post mortem, base point lesions are not a profound factor in DNA degradation. As would be expected, we consistently observed significant differences between transversions and transitions across the time periods and tissues (Table 7). However, we found no significant difference between type 1 and type 2 transitions at any time point. Therefore we postulate that most aDNA point degradation occurs either during putrefaction or in the long-term exposure to environmental conditions, or both, as we found no significant degradation over the desiccation period.

## Conclusions

Mummification can occur under a wide range of desiccating or anoxic conditions, but only mummification by desiccation generally provides high quality DNA. The results of this pilot study in artificial salt mummification indicate that salt desiccation is an effective method for medium to long-term preservation of human tissues at the molecular level. Overall, DNA preservation in salt-desiccated skin and muscle was observed to be excellent, with damage-free, amplifiable DNA fragments in excess of 4000 bp nearly one year post mortem. Among tissues, preservation was greater in skin, most likely because this tissue was in direct contact with the natron salt and thus was more efficiently dehydrated than interior tissues, such as muscle. Although DNA fragmentation increased over the course of the experiment, with noticeably higher fragmentation in muscle than in skin, no significant increases in miscoding lesions were observed in either skin or muscle. These results suggest that under salt desiccation little nucleotide damage occurs, and DNA strand breaks are slowed in proportion to salt proximity. Natron mummification results in remarkable DNA preservation and likely contributes to the long-term survival of DNA in a range of natural and artificial mummified tissues.
